# "So-Called" Aseptic Surgery

**Published:** 1903-02-14

**Authors:** 


### HOSPITAL CLINICS AND MEDICAL PROGRESS.

### "SO-CALLED" ASEPTIC SURGERY.

AT the annual meeting of the Harveian Society the retiring President, Mr. Watson Cheyne, F.R.S., made some critical remarks upon what he describes as "this most modern development of Listerism, the so-called 'aseptic surgery.'" He says that he uses the epithet "so-called" because, as at present employed, this term "aseptic surgery" is in his opinion an entire misnomer. More than twenty years ago he used the term aseptic surgery in contrast with the term antiseptic surgery, but with a different meaning from that in which these terms are employed to-day. Antiseptic surgery he defined as surgery directed against sepsis, or at any rate against septic organisms which had already entered a wound, the aim of antiseptic surgery being therefore the eradication of these organisms from the wound. By the term aseptic surgery, on the other hand, he implied surgery in which means were taken to prevent the entry of organisms into the wound. Aseptic surgery or the production of an aseptic wound could be carried out in a great variety of ways, but so long as the end was obtained—namely, the prevention of sepsis in the wound—the various methods came under the heading of aseptic surgery. At the present time, however, these terms are being employed quite differently, antiseptic surgery being used to indicate methods in which antiseptics are employed and aseptic surgery where no antiseptics are employed. This is a totally different conception, and one which he believes does not touch the root of the matter, which is not the use of particular methods but the prevention of the entrance of bacteria. To label the methods according to the materials employed is apt, he says, to lead the surgeon to overlook the principle involved. Moreover, aseptic surgery as at present defined—that is without the use of antiseptics—is impossible, for antiseptics are the only means that can be employed for the most important of all the precautions which are required in providing for an aseptic wound—namely the disinfection of the skin both of the patient and the surgeon. Referring to the difficulty which seems now to be experienced by so many surgeons in sterilising sutures, whether catgut or silk, Mr. Watson Cheyne says that to him this is a most remarkable state of affairs, of which he has practically no experience. He uses catgut as frequently as he formerly did, and has no hesitation in uniting muscular planes with silk and leaving the stitches buried, and although no one is infallible, and occasionally asepsis is not obtained, this is so extremely rare that it is quite evidently due to some oversight in the methods employed, and not to anything wrong with the material. From his own experience and from the experience of others who have been trained up in Listerian methods, he cannot admit that when infection occurs in a wound it is necessarily, or even probably, due to ligatures and stitches. That when a wound becomes infected during an operation the stitches, being dead material in which infective organisms can multiply, must necessarily be extruded from the wound, and that such wounds will not heal until these deep stitches have come out, is obvious; but to assume that therefore it is the sterilisation of the stitches that has been at fault is an entirely illogical conclusion. These troubles as regards disinfection of the ligatures and stitch abscesses have essentially arisen, he says, since the introduction of methods from which antiseptics have been more or less completely excluded. After describing the very elaborate precautions which are essential if "aseptic surgery" without antiseptics is to be safely employed, he adds that although the method is one with which he has no fault to find in theory, or if carried out by trained bacteriologists, it is not one which can by any possibility be recommended for general use, and that he does not think that students, who will not necessarily have all the arrangements at hand in after life, should be instructed in it as the only or chief method which they should employ. No doubt if all this sort of treatment is really necessary, nothing further can be said than that everyone must try to carry it out; but it is not really necessary, because those who employ antiseptics in a limited manner on the Listerian principles, obtain equally good results—indeed more constant results; and one cannot get a better result than rapid and constant healing by first intention without any local or general disturbance. Mr. Watson Cheyne believes that if this so-called aseptic treatment is continued, a time will come when the frequency of sepsis in wounds will be very considerable, and when the thoroughness that is characteristic of surgery at the present time will become diminished, as indeed is already happening in various ways, such as the careful stitching up of the deeper parts of abdominal wounds. The intelligent use of antiseptics saves a great deal of trouble, makes the result more certain, and does not interfere in any way with the after progress of the wound. It is evident that it is the greater certainty and constancy of the results under the use of antiseptics that more particularly influences Mr. Watson Cheyne in his verdict.

